# Supplementary material for: “Can Do” vs. “Do Do” in Older Adults: A Cross-Sectional Analysis of Sensor-Derived Physical Activity Patterns
Source: Sensors (Basel). 2023 Feb 7;23(4):1879. doi: 10.3390/s23041879 (PMC9959454; doi:10.3390/s23041879)
Supplement: Supplementary file 1 [file sensors-23-01879-s001.zip › sensors-2144677-supplementary.pdf]

Table S1. Effect sizes by the comparison of the PA variables in the four quadrants

Variable	Gruppenvergleich	n	Z	p	ES	Interpretation
p95 cadence (in steps per min).	Can do don't do - Can't do do do	116	0.63	0.53	0.06	low
	Can do do do - Can do don't do	126	0.50	0.61	0.04	low
	Can do don't do - Can't do don't do	142	-2.43	0.02	0.20	low
	Can do do do - Can't do do do	152	1.31	0.19	0.11	low
	Can't do don't do - Can't do do do	168	-2.04	0.04	0.16	low
	Can do do do - Can't do don't do	178	3.54	<0.01	0.27	low
mean cadence (in steps per min)	Can do don't do - Can't do do do	116	-4.36	<0.01	0.40	moderate
	Can do do do - Can do don't do	126	4.44	<0.01	0.40	moderate
	Can do don't do - Can't do don't do	142	-1.01	0.31	0.09	low
	Can do do do - Can't do do do	152	-0.03	0.98	<0.01	low
	Can't do don't do - Can't do do do	168	-6.49	<0.01	0.50	large
	Can do do do - Can't do don't do	178	6.71	<0.01	0.50	large
walking duration lower 3 METs (in min).	Can do don't do - Can't do do do	116	-7.08	<0.01	0.66	large
	Can do do do - Can do don't do	126	6.42	<0.01	0.57	large
	Can do don't do - Can't do don't do	142	0.65	0.52	0.05	low
	Can do do do - Can't do do do	152	-0.95	0.34	0.08	low
	Can't do don't do - Can't do do do	168	-7.89	<0.01	0.61	large
	Can do do do - Can't do don't do	178	7.16	<0.01	0.54	large
walking duration greater or equal 3 METs (in min).	Can do don't do - Can't do do do	116	-6.83	<0.01	0.63	large
	Can do do do - Can do don't do	126	7.77	<0.01	0.69	large
	Can do don't do - Can't do don't do	142	-1.82	0.07	0.15	low
	Can do do do - Can't do do do	152	0.88	<0.01	0.07	low
	Can't do don't do - Can't do do do	168	-10.43	<0.01	0.80	large
	Can do do do - Can't do don't do	178	11.77	<0.01	0.88	large

Percentage walking duration greater or equal 3 METs (in %).	Can do don't do - Can't do do do	116	1.76	<0.01	0.16	low
	Can do do do - Can do don't do	126	-3.63	<0.01	0.32	moderate
	Can do don't do - Can't do don't do	142	2.86	<0.01	0.24	low
	Can do do do - Can't do do do	152	-2.09	<0.01	0.17	low
	Can't do don't do - Can't do do do	168	5.45	<0.01	0.42	moderate
	Can do do do - Can't do don't do	178	-7.91	<0.01	0.59	large
maximum continuous number of steps	Can do don't do - Can't do do do	116	-3.45	<0.01	0.32	moderate
	Can do do do - Can do don't do	126	5.01	<0.01	0.45	moderate
	Can do don't do - Can't do don't do	142	-1.98	0.05	0.17	low
	Can do do do - Can't do do do	152	1.68	0.09	0.14	low
	Can't do don't do - Can't do do do	168	-6.50	<0.01	0.50	large
	Can do do do - Can't do don't do	178	8.56	<0.01	0.64	large
maximum walking interval length (in sec).	Can do don't do - Can't do do do	116	-3.64	<0.01	0.34	moderate
	Can do do do - Can do don't do	126	4.89	<0.01	0.44	moderate
	Can do don't do - Can't do don't do	142	-1.13	<0.01	0.09	low
	Can do do do - Can't do do do	152	1.32	0.19	0.11	low
	Can't do don't do - Can't do do do	168	-5.74	<0.01	0.44	moderate
	Can do do do - Can't do don't do	178	7.39	<0.01	0.55	large
average walking interval length (in sec).	Can do don't do - Can't do do do	116	-3.27	<0.01	0.30	moderate
	Can do do do - Can do don't do	126	4.57	<0.01	0.41	moderate
	Can do don't do - Can't do don't do	142	-1.35	0.18	0.11	low
	Can do do do - Can't do do do	152	1.39	0.16	0.11	low
	Can't do don't do - Can't do do do	168	-5.54	<0.01	0.43	moderate
	Can do do do - Can't do don't do	178	7.25	<0.01	0.54	large
number of walking intervals.	Can do don't do - Can't do do do	116	-7.47	<0.01	0.69	large
	Can do do do - Can do don't do	126	7.19	<0.01	0.64	large

	Can do don't do - Can't do don't do	142	-0.18	0.86	0.02	low
	Can do do do - Can't do do do	152	-0.53	0.60	0.04	low
	Can't do don't do - Can't do do do	168	-9.32	<0.01	0.72	large
	Can do do do - Can't do don't do	178	9.10	<0.01	0.68	large
Percentage number of walking intervals length <10s .	Can do don't do - Can't do do do	116	6.13	<0.01	0.57	large
	Can do do do - Can do don't do	126	-5.70	<0.01	0.51	large
	Can do don't do - Can't do don't do	142	-1.45	0.15	0.12	low
	Can do do do - Can't do do do	152	0.66	0.51	0.05	low
	Can't do don't do - Can't do do do	168	5.80	<0.01	0.45	moderate
	Can do do do - Can't do don't do	178	-5.31	<0.01	0.40	moderate
Percentage number of walking intervals length <20s .	Can do don't do - Can't do do do	116	3.95	<0.01	0.37	moderate
	Can do do do - Can do don't do	126	-4.31	<0.01	0.38	moderate
	Can do don't do - Can't do don't do	142	0.62	0.54	0.05	low
	Can do do do - Can't do do do	152	-0.29	0.77	0.02	low
	Can't do do do - Can't do don't do	168	5.53	<0.01	0.43	moderate
	Can do do do - Can't do don't do	178	-6.06	<0.01	0.45	moderate
Percentage number of walking intervals length <60s .	Can do don't do - Can't do do do	116	-0.22	0.82	0.02	low
	Can do do do - Can do don't do	126	-0.14	0.89	0.01	low
	Can do don't do - Can't do don't do	142	2.16	0.03	0.18	low
	Can do do do - Can't do do do	152	-0.42	0.67	0.03	low
	Can't do do do - Can't do don't do	168	2.22	0.03	0.17	low
	Can do do do - Can't do don't do	178	-2.76	0.01	0.21	low

n= number of participants; z= z-value; p= p-value ES= effect size calculated by z/square root(n); *ES: 0.1-0.3=low. 0.3 - 0.5=moderate. greater than 0.5=high
